# The application of synthetic antibacterial minerals to combat topical infections: exploring a mouse model of MRSA infection

**DOI:** 10.1038/s41598-024-52082-8

**Published:** 2024-01-19

**Authors:** Keith D. Morrison, Meghan B. Reiss, Tanya D. Tanner, Travis R. Gollott, Gabriela G. Loots, Nicole M. Collette

**Affiliations:** 1 Nuclear and Chemical Sciences Division, Physical and Life Sciences, Lawrence Livermore National Laboratory, Livermore, CA USA; 2 Biosciences and Biotechnology Division, Physical and Life Sciences, Lawrence Livermore National Laboratory, Livermore, CA USA; 3 Department of Orthopaedic Surgery, University of California Davis Health, Sacramento, CA USA

**Keywords:** Antimicrobials, Biogeochemistry

## Abstract

The development of new antibiotics has stalled, and novel strategies are needed as we enter the age of antibiotic resistance. Certain naturally occurring clays have been shown to be effective in killing antibiotic resistant bacteria. However, these natural clays are too variable to be used in clinical settings. Our study shows that synthetic antibacterial minerals exhibit potent antibacterial activity against topical MRSA infections and increase the rate of wound closure relative to controls. The antibacterial minerals maintain a redox cycle between Fe^2+^/Fe^3+^ and the surfaces of pyrite minerals, which act as a semiconductor and produce reactive oxygen species (ROS), while smectite minerals act as a cation exchange reservoir. Acidic conditions are maintained throughout the application of the hydrated minerals and can mitigate the alkaline pH conditions observed in chronic non-healing wounds. These results provide evidence for the strategy of ‘iron overload’ to combat antibiotic resistant infections through the maintained release of Fe^2+^ and generation of ROS via distinct geochemical reactions that can break the chronic wound damage cycle.

## Introduction

The discovery of antibiotics by Alexander Fleming in the 1920s changed the world and ushered in a new era of health and longevity never before seen by humanity. However, antibiotic resistance has been continually jeopardizing the security and safety afforded by antibiotics. Antibiotic resistance in microbial infections has been increasing since the discovery of antibiotics, making the antibiotic resistance era an ever-present threat to global modern lives^1–8^. Traditional antibiotics are organic molecules that are generated by microorganisms in the environment to kill other microbial species that are competing for the nutrients required for cell growth^1,4,5^. The evolution of antibiotic synthesis and resistance in microorganisms began well before the discovery and application of antibiotics for human health applications. However, the widespread over-use of antibiotics on a global scale has accelerated the evolution and spread of antibiotic resistance genes in clinical and natural environments^1,2,4–6^. Unfortunately, the discovery of new antibiotics has mostly stalled and traditional antibiotic discovery techniques have not produced new drugs in over 30 years^5,6^. Additionally, all the current new antibiotics discovered have chemical structures and modes of action that are analogous to current antibiotics^5,6^. With the discovery of novel antibiotics in decline, research must also investigate other natural processes to combat infections and mortality in the era of antibiotic resistance.

The current estimated global deaths from antibiotic resistance are of grave concern, with 4.95 million (M) people dying per year from illnesses where antibiotic resistance played a role and 1.27 M deaths per year directly attributed to antibiotic resistant infections^9–11^. Currently, deaths from antibiotic resistant infections have surpassed deaths from HIV/AIDS [864 thousand (K)] and malaria (643 K)^9^, with global annual deaths anticipated to reach 10 M by the year 2050, outpacing current cancer deaths (8.2 M)^11^. Overall, only six pathogens are associated with the majority of global deaths from antibiotic resistance^12^. The number one killer was *Escherichia coli*, followed by *Klebsiella pneumoniae, Staphylococcus aureus, Streptococcus pneumoniae, Acinetobacter baumannii, and Pseudomonas aeruginosa *^12^. *Methicillin-resistant Staphylococcus Aureus* infections represent a significant threat to public health and spread through community and hospital acquired infections^13–15^, resulting in 178 K deaths per year globally and over 120 K of those deaths resulting from methicillin resistance^12^. While MRSA infections have been declining in the United States^10^, the wide spread use of intravenous drugs coupled to homelessness and poor living conditions may have the potential to exacerbate our current efforts to prevent the spread of infections^11,16^. The stalled discovery of antibiotics^1,5,6^ stems from both scientific and economic factors. The lack of profitability, incentives and investments lead to stagnant growth of new antibiotics. Additionally, many of the current strategies for antimicrobial discovery still rely on the discovery of traditional antibiotic compounds that are susceptible to the evolution of resistance on short time scales (< 3 years)^4,17^. Antimicrobial compounds that attack multiple cellular systems (DNA, RNA, proteins, lipids) may offer some reprieve from the single point genetic mutations that can establish antibiotic resistance in bacteria.

Metals have been used throughout history to combat microbial infections, with the application of Ag, Cu receiving the most attention^18–22^. Iron (Fe) represents a double-edged sword for bacterial growth, as it is a limiting nutrient for growth but can generate toxicity if excess cellular uptake occurs^20,21^. Excess Fe in wounds is traditionally viewed as a negative outcome for wound healing as it accelerates bacterial growth and the formation of biofilm infections while promoting toxicity from reactive oxygen species (ROS) generation^23–29^. The chelation of excess Fe in wounds has been used as a strategy to limit bacterial growth and promote wound healing, but success with this approach has been limited^30,31^. The discovery of natural clay mineral deposits with Fe based antibacterial activity revealed that geochemical reactions that maintain ferrous iron (Fe^2+^) and ROS can serve as potent antimicrobials^32–36^. These antibacterial zones in natural deposits are composed of smectite clays and iron sulfides (pyrite) that experience a cascade of redox reactions as they achieve a new chemical equilibrium^32–34,36,37^. This strategy of ‘mineral iron-overload’ to cure antibiotic resistant infections appears counter intuitive when compared to the common assumptions that excess iron and ROS are associated with delayed healing and negative outcomes in chronic wounds^23,24,26,27^. However, anecdotal evidence from natural clays^38^ and limited animal model MRSA infections reveals that these antibacterial mineral mixtures may promote wound healing while killing antibiotic resistant infections^32–36,39,40^ The clinical application of these natural mineral deposits has proved difficult due to the large variations in mineralogy and antibacterial activity along with the presence of toxic metal impurities that produce variable outcomes in wound care^32,34,39,40^. The recent synthesis of antibacterial minerals with properties that mimic the natural analogues proved successful at killing the ESKAPE pathogens (*Enterococcus* sp.,* Staphylococcus aureus, Klebsiella pneumoniae, Acinetobacter* sp.,* Pseudomonas aeruginosa* and *Enterobacter* sp.) providing a chemically pure and consistent dose that can be scaled for pharmaceutical production^7,41^. Furthermore, the synthetic antibacterial mineral systems can be tuned to have different reaction rates that can maintain Fe^2+^ and ROS production at specific concentrations from days to weeks when hydrated^41^. This tuning and control of concentration and reactivity is not possible with the application of metal solutions alone. Materials that control the solubility and speciation of metal solutions are needed to make a clinically effective metal-based antimicrobial. The effective treatment of treat topical *methicillin-resistant Staphylococcus aureus* (MRSA) infections has yet to be proven with synthetic antibacterial mineral systems, limiting avenues for clinical and pharmaceutical applications. This study explores the use of synthetic antibacterial minerals to combat topical MRSA infections in a mouse model.

### Clinical problem addressed

Chronic non-healing wounds infected with multi-drug resistant bacterial infections represent a growing global health crisis and increase the risk of amputation and mortality in individual suffering from diabetes, neuropathy or vascular disorders^1,2,7,10,42,43^. Losing the ability to treat common bacterial infections with antibiotics is predicted to result in significant loss of life and will place strain on global economies^10,14^. Synthetic antibacterial mineral formulations that recreate the antimicrobial activity of natural mineral deposits are a promising solution for treating antibiotic resistant topical infections. Establishing the efficacy of these mineral formulations for treating topical MRSA infections and promoting wound closure is required before these novel antimicrobials can be adopted in clinical settings. Our research shows that synthetic antibacterial minerals can successfully eliminate MRSA biofilms in topical wounds and increase wound closure rates. The exploration of synergistic mineral based geochemical reactions for antimicrobial applications is still in its infancy and further research is needed to transition this technology into clinical settings. These minerals may be compatible with a variety of self-assembled nanomaterials, polymers and 3D printed wound dressings that can be used to control and tune novel antibacterial mechanisms^44^.

## Materials and methods

### Antibacterial mineral synthesis

The synthetic antibacterial minerals consist of synthetic smectite clays and iron-sulfide (pyrite) that are synthesized with specific particle size, cation exchange capacity and reactivity that mimics the antibacterial activity of the antibacterial clay deposits discovered in nature^32–34,36,40,41^. The synthesis of antibacterial minerals was achieved following the methods of Morrison et al., 2022^41^. Synthetic smectite clays with a hectorite elemental composition were generated by reacting fumed silica nanoparticles, Mg(OH)_2_ and LiF in a hydrothermal pressure reactor at 200 °C for 5 days. The resulting smectite clays were placed in dialysis tubing and excess cations were rinsed away in a deionized water bath. Synthetic pyrite micro-spheres were synthesized using a solution of polyvinylpyrrolidone, polysulfides and ferrous chloride that was heated for 48 h in a hydrothermal reactor. The pyrite micro-spheres were further purified with acid and solvent rinses. The final mineral powders were freeze dried and stored in a vacuum desiccator.

Synthetic antibacterial mineral mixtures were created that contained 95 wt.% smectite and 5 wt.% pyrite. These mineral mixtures were then exchanged with 30 mM solutions of ferrous sulfate at a mineral concentration of 10 mg/mL. The mixtures were placed in an ultrasonic bath for 10 min, centrifuged to pellet the minerals, resuspended in ethanol and centrifuged to pellet the minerals. The Fe^2+^ exchanged mineral mixtures were freeze dried and stored in a vacuum desiccator. The freeze dried antibacterial mineral mixtures were autoclaved before application to topical MRSA infections.

### Dose and extended release of ferrous iron and hydrogen peroxide

We measured the release of Fe^2+^ and H_2_O_2_ from 3 antibacterial mineral slurries with concentrations ranging from 50, 75 and 100 mg/mL in tryptic soy broth (15 g/L), lauria broth (10 g/L), RPMI media or de-ionized water over 72 h. The minerals consisted of 4 separate batches of synthetic antibacterial mineral mixtures prepared as described in the previous section. All Fe^2+^ and H_2_O_2_ results were normalized to the mineral concentration and reported as mM (Fe^2+^ or H_2_O_2_) per gram of mineral. This was done to determine if a range of mineral concentrations produced the same dose response per gram of minerals. Mineral concentrations above 100 mg/mL prevented the accurate measurement of H_2_O_2_ in solution due to the presence of un-chelated Fe^2+^ (when mixed with the reagent buffer) that catalyzes the decomposition of H_2_O_2_ into hydroxyl radicals. Quantification of Fe^2+^ and H_2_O_2_ in mineral leachate solutions was achieved using 1,10-phenanthroline and horseradish peroxidase/leuco crystal violet, respectively, following the methods of Morrison et al., 2022^41^. Additionally, pH and Eh measurements were measured on all mineral suspensions.

### Thermodynamic modeling of metal speciation and mineral stability fields

The speciation modeling and mineral stability pH and Eh diagram for the Fe–S–O–H system were calculated using Geochemist’s Workbench V14.0 using the MINTEQ thermodynamic database of equilibrium constants. The Fe and S stability fields were calculated from infinite dilution up to 2 mM concentrations to represent the transition to antibacterial levels of Fe.

### Animal model

These studies were carried out in strict accordance with the recommendations in the *Guide for the Care and Use of Laboratory Animals* and the National Institute of Health. All efforts were made to minimize suffering of animals. All animals were housed in ABSL2 conditions in an AAALAC-accredited facility, and the protocol was approved by the LLNL Institutional Animal Care and Use Committee (IACUC), which includes ethics in evaluation of protocols and adherence to the ARRIVE guidelines. Barrier-housed, specific pathogen-free SKH-1 hairless mice (Elite health status, Crl:SKH1*-Hr*^*hr*^) were obtained from Charles River Laboratories (CRL, catalog #477SKH1-E), and acclimated for one week prior to experiments. *Methicillin-Resistant Staphylococcus aureus* (MRSA) and animal use was also approved by the Institutional Biosafety Committee (IBC).

### MRSA infections in a mouse model

A 2 cm^2^ skin wound was scraped on the backs of 6–8 week old SKH-1 hairless mice using a scalpel. Following this procedure, the skin was visibly damaged and characterized by reddening and glistening but did not bleed. The wounds were prepared on mice anesthetized using intraperitoneal injections of 0.05 ml per 25 g of body weight with a mixture containing 21 mg ketamine, 2.4 mg xylazine and 0.3 mg acepromazine. Anesthetic plane was monitored by toe pinch, respiration rate, and skin color, and the animals were placed on an infrared heating pad set slightly above body temperature for the duration of the procedure. The skin was scrubbed using alternating chlorhexidine surgical scrub and water, finishing with 70% ethanol rinse and allowed to air dry. Mice were given pre-operative buprenorphine at a 0.1 mg/kg concentration as a subcutaneous injection. The wounds were next inoculated with 20 μL 5 × 10^8^ CFU/mL MRSA (strain ATCC 43,300) that was grown to log phase in tryptic soy broth (30 g/L). MRSA was pipetted directly onto the fresh wound and spread with the broad side of the pipette tip across the entire wound site until it was adsorbed into the wound, and the surface looked dry (n = 10 per group; both males and females were examined in all experiments). The inoculum was prepared by spinning bacteria at 800 × g to pellet. Growth media was removed, and bacteria were pelleted twice and resuspended in isotonic NaCl (0.9 wt.%) (USP) to 5 × 10^8^ CFU/mL for dosing. Control wounds were “inoculated” with sterile isotonic solution (n = 10 per group). The wound was then covered with a moist non-stick wound pad, cut to size, and covered with Tegaderm (3 M), which is a clear adhesive sheet bandage material that is air permeable, but not liquid permeable, for 48 h to allow the MRSA infection to form a biofilm. Once the biofilms were established, a hydrated mineral poultice was applied at a concentration of 400 mg/mL (sterile mineral/isotonic solution) and 200 μL was be pipetted onto the wound (n = 10 per group). The wounds were then covered with a Tegaderm bandage as described above. The wounds were monitored for a total of 8 days, with minerals applied at day 2 (after MRSA biofilm formation) and day 4. The bandage was observed every day, and bandages changed as need to manage bandage integrity. Animals were anesthetized under 4–5% inhaled isoflurane and maintained at 1–3% isoflurane on a nosecone for wound management. Wound areas were quantified using ImageJ-Fiji^45^ and reported as the percentage decrease in area (cm^2^) after 8 days, relative to the initial wound area.

Wound bacterial CFU measurements were performed at the terminal timepoint for the study (Day 8). Briefly, the wound area of infected and control animals was excised and homogenized in 0.5 ml fresh sterile phosphate-buffered-saline in 2 ml tubes that contained 2.8 mm hard-tissue ceramic beads (OMNI International, catalog# 19-628-3). Tissues were homogenized for two rounds of 30 s with a cooling period between homogenizations on an OMNI Bead-Ruptor 12 (OMNI International). Serial dilutions were made, and the homogenates were plated on Luria Broth (LB) agar plates containing no antibiotics (Teknova). Plates were incubated overnight at 37 degrees and colonies were counted after 24 and 48 h to calculate total CFU per tissue. Results were plotted with either standard error or as box plots for the 25th to 75th percentiles with whiskers extending 1.5 times the interquartile range. Statistical significance was assessed using one-way analysis of variance (ANOVA) and presented as probability values (*P-*values), with values < 0.005 considered statically significant.

## Results and discussion

### Synthetic antibacterial minerals and MRSA wound closure

**Figure 1:**
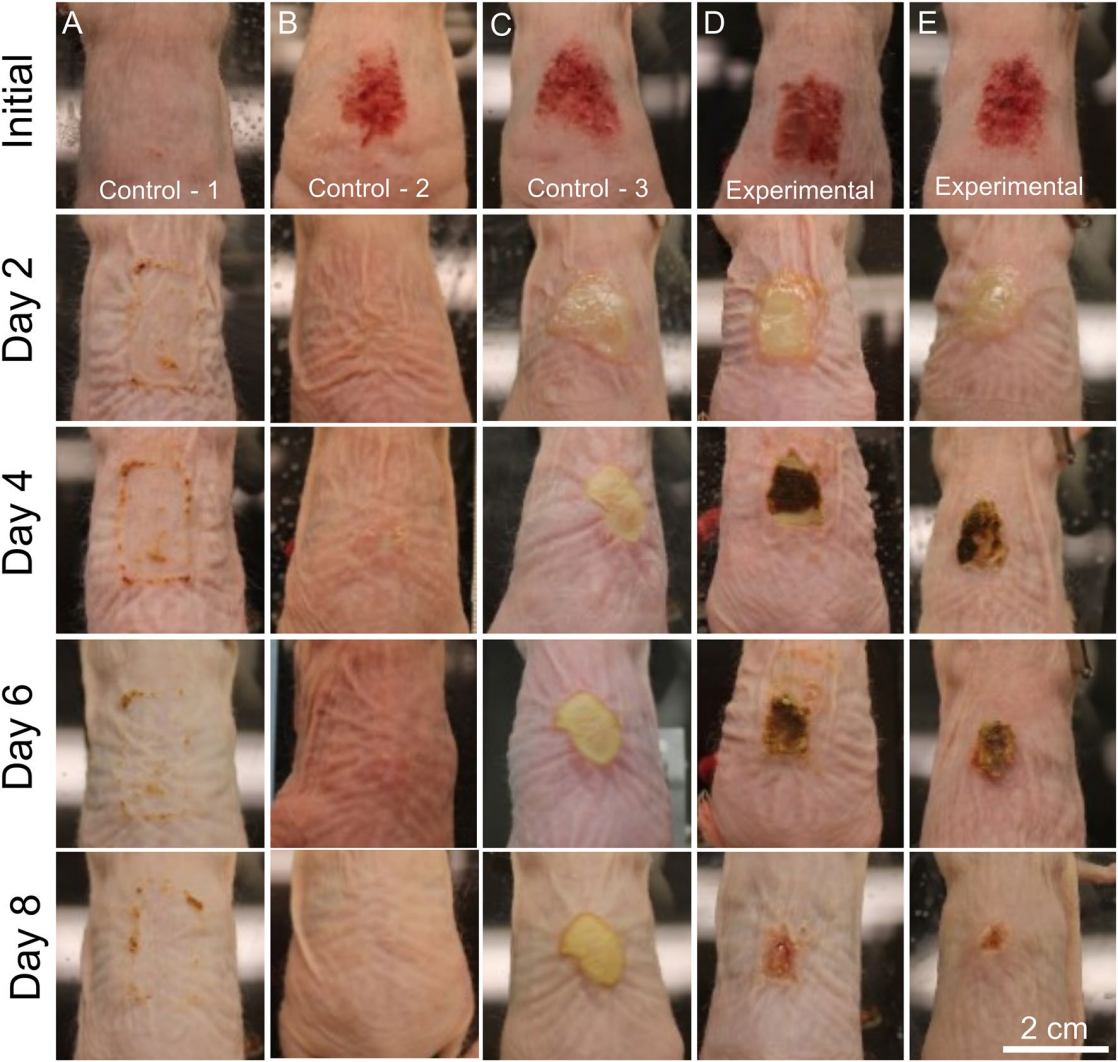
Representative images of each treatment group. MRSA infections treated with antibacterial minerals were examined over 8 days. Antibacterial minerals (control 1; minerals only, no wound) applied to intact skin over 8 days (application at days 1 and 2) (**A**). Scalpel scrape wound (control 2; wound only, no infection or minerals) (**B**); MRSA-infected untreated mice (control 3: MRSA infection only) (**C**); MRSA infections treated with 400 mg/mL doses of antibacterial minerals (experimental group) applied at day 2 and day 4 animal with least healing (**D**) and with most healing (**E**) observed.

The scalpel scrape wounds produced red slightly moist wounds with the top layer of epidermis removed, producing a wound that extended partially into the dermis (Fig. 1). The wounds did not extend into the hypodermis as there were no signs of subcutaneous fat or prolonged bleeding from blood vessel disruption. The application of synthetic antibacterial minerals to intact skin with no wound or MRSA infection resulted in minor contact dermatitis along the edges of the gauze and Tegaderm bandage (Fig. 1A). The skin irritation occurred as minerals migrated to the periphery of the Tegaderm bandage and accumulated where the adhesive maintained contact with the skin and remained moist. The control wounds with no MRSA infections showed a rapid reduction in reddening and a thin glistening scab layer formed, indicating a steady transition from the inflammation to proliferation stages of wound healing after 2 days (Fig. 1B). On days 4 and 6, wound remodeling can be observed and by day 8 the control wounds showed no signs of injury (Fig. 1B). The MRSA infected control wounds produced visible biofilms with a shiny yellow surface that covered the extent of the scalpel scrape wound after 2 days (Fig. 1C). The MRSA control wounds maintained a biofilm infection throughout the 8 days of reaction, indicating a robust and persistent wound model. The application of antibacterial minerals to the MRSA biofilms occurred on day 2 and produced a dark brown mineral coating on the biofilms that led to scabbing by day 4 (Fig. 1D,E). The second dose of minerals was applied on day 4 and resulted in increased wound closure and scabbing, while the MRSA control wound showed no signs of healing. After 8 days the wound scab fell off as the wound diameter continued to decrease and the MRSA infection cleared (Fig. 1D,E). The visual changes to the MRSA wound environment during the application of antibacterial minerals shows that these formulations can be applied topically to rapidly kill bacterial infections and promote wound healing.

**Figure 2:**
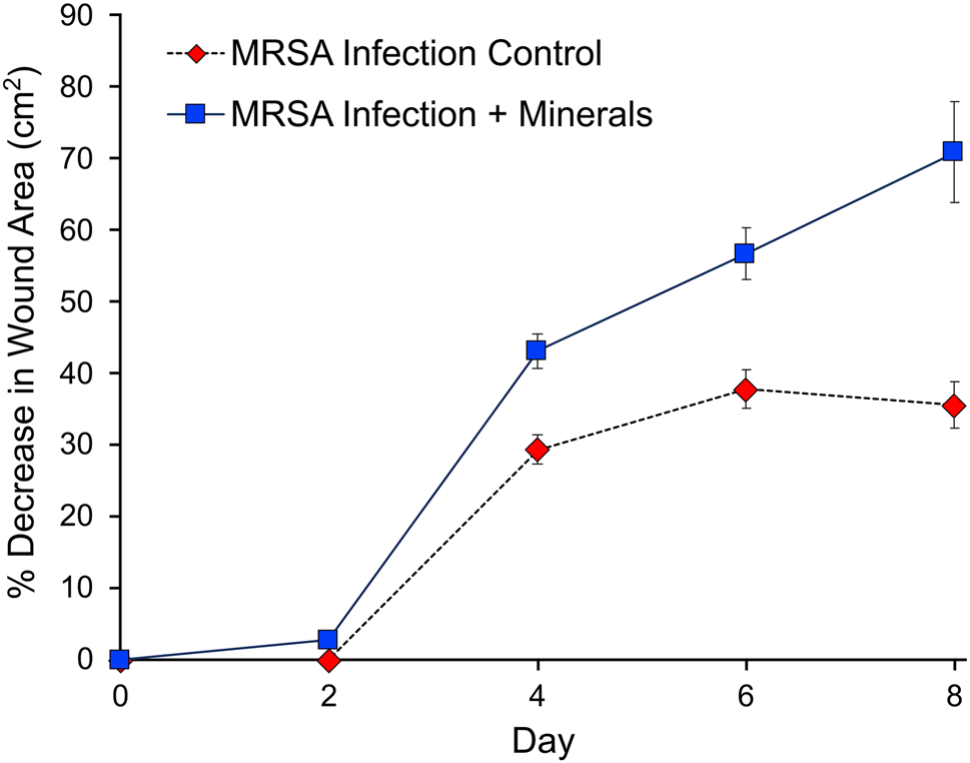
Percent decrease in wound area in MRSA infection controls and mineral treated samples measured over 8 days.

Wound areas were measured over the 8 day experiment and reported as the percentage decrease in wound area (cm^2^) (Fig. 2). After the MRSA infection was established on day 2 of the reaction, the antibacterial minerals were applied. The wound areas in the mineral treated infections showed a 71.0% (± 6.9%) average decrease in wound area after 8 days of treatment (Fig. 2). The wound area decreases in control MRSA infections plateaued on days 4–8, while the mineral treated wounds saw a continued decrease in wound area. The control MRSA infections showed a 35.6% (± 3.2%) decrease in wound area after 8 days, with the majority of wounds still showing signs of MRSA biofilms (Figs. 1 and 2).

### Wound bacterial cell concentrations

**Figure 3:**
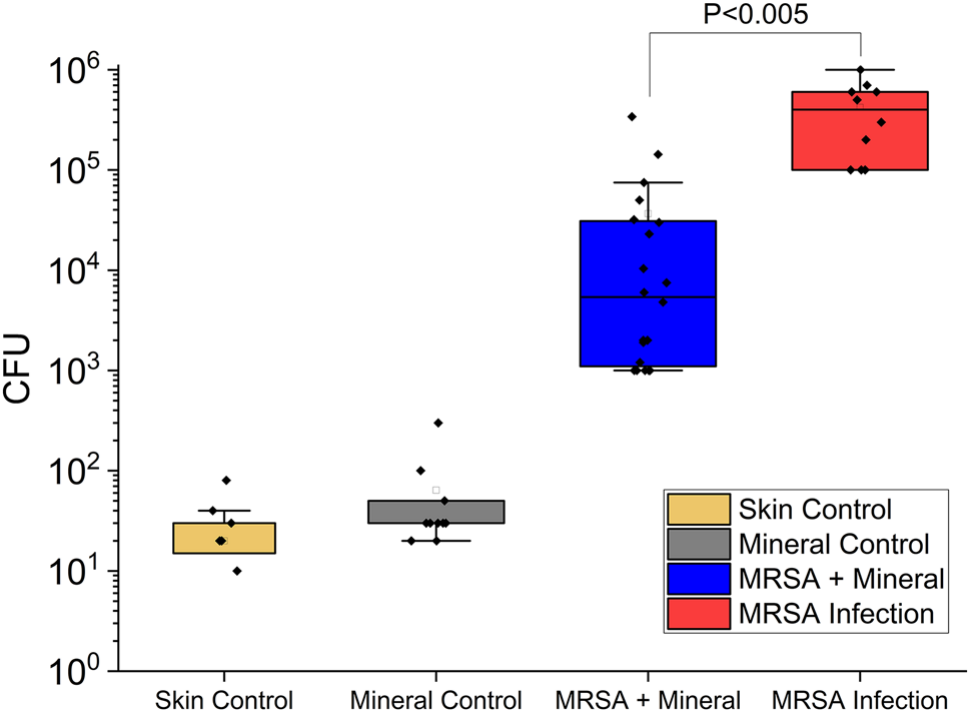
Box plots of bacterial colony forming units (CFU) (after 8 days) from mice with no wound or infections (Skin Control), no wound or infections plus mineral exposure with two mineral applications over 8 days (Mineral Control), MRSA infections treated with two mineral applications over 8 days (MRSA + Mineral) and an untreated control MRSA infection with no mineral application. The probability values between the MRSA + mineral and MRSA infection groups have P-values < 0.005, indicating a statistically significant difference in the synthetic antibacterial mineral treated infection.

Wound bacterial cell concentrations were measured at the end of the 8-day trial by excising the wound tissues, followed by homogenization in a bead shaker. The homogenized tissues were plated for CFU counting. At the end of the 8-day trial the mouse skin controls with no wound and no wound plus minerals showed low total tissue bacterial loads with tens to hundreds of cells measured (Fig. 3). The MRSA infected wounds had higher bacterial CFU counts, with the MRSA infections having median cell concentrations of 5.63 log_10_ (CFU) at the end of 8 days. The MRSA infections treated with minerals had median bacterial cell concentrations of 3.89 log_10_ (CFU) representing a 1.74 log_10_ reduction in wound bacterial cells. This reduction represents a 98.2% reduction in median wound bacterial cell loadings, which represents a statistically significant (*P* = 0.0031) reduction of topical MRSA biofilm infections. The bacterial CFUs measured in MRSA infections treated with minerals produced more scattered results when compared to the MRSA infection and controls (Fig. 3).

Three outlier datapoints were present in the mineral treated infections that fell above the upper whisker in the box plot outside the 4th quartile group. Additionally, the upper and lower quartiles (representing 50% of the observed data) in the mineral treatments span a larger range when compared to the MRSA infection control. The mineral treated mice showed a 1.5 log_10_ range in the upper and lower quartiles while the MRSA infection control had a 0.78 log_10_ range (Fig. 3). The greater spread in bacterial CFUs in the mineral treated MRSA infections was likely due to differences in the application of the aqueous mineral suspensions with gauze. Direct contact of the antibacterial minerals with the entire MRSA biofilm infection is needed throughout the duration of treatment and more advanced hydrogel, biopolymer or 3D printed matrices may provide more consistent patient outcomes when applied in a clinical setting.

### Geochemistry of synthetic antibacterial minerals

The release of Fe^2+^ and generation of H_2_O_2_ was measured using multiple batches of antibacterial minerals synthesized over a 2-year period. This was done to determine if the mineral synthesis and formulation procedures resulted in a product with reproducible reactivity and antibacterial properties. The final antibacterial mineral formulations were prepared (Fe^2+^ exchanged and autoclaved) the week before testing as described in the methods section. Antibacterial mineral concentrations ranged from 50, 75 and 100 mg/mL and bacterial growth and cell culture media (TSB, LB, RPMI or DIW) were used to determine if reproducible doses could be achieved in a range of cation, anion and organic substrates. Previous studies reveal that a pulse of Fe^2+^ and H_2_O_2_ occur when natural or synthetic antibacterial minerals are hydrated for antimicrobial applications^33,34,41^. These studies report the concentrations of mineral suspensions (mg/mL) along with the associated metal or ROS release. In this study we wanted to determine if concentrations of Fe^2+^ and H_2_O_2_ released over time are proportional to the mineral mass over a wide dose range. It is important that different doses of the compound have similar reactivity, metal release, ROS generation and pH buffering. By normalizing antibacterial mineral doses to the added mass and measuring Fe^2+^, H_2_O_2_ and pH in a range of buffering and chelating solutions, we were able to determine if consistent antibacterial mineral doses could be applied.

**Figure 4:**
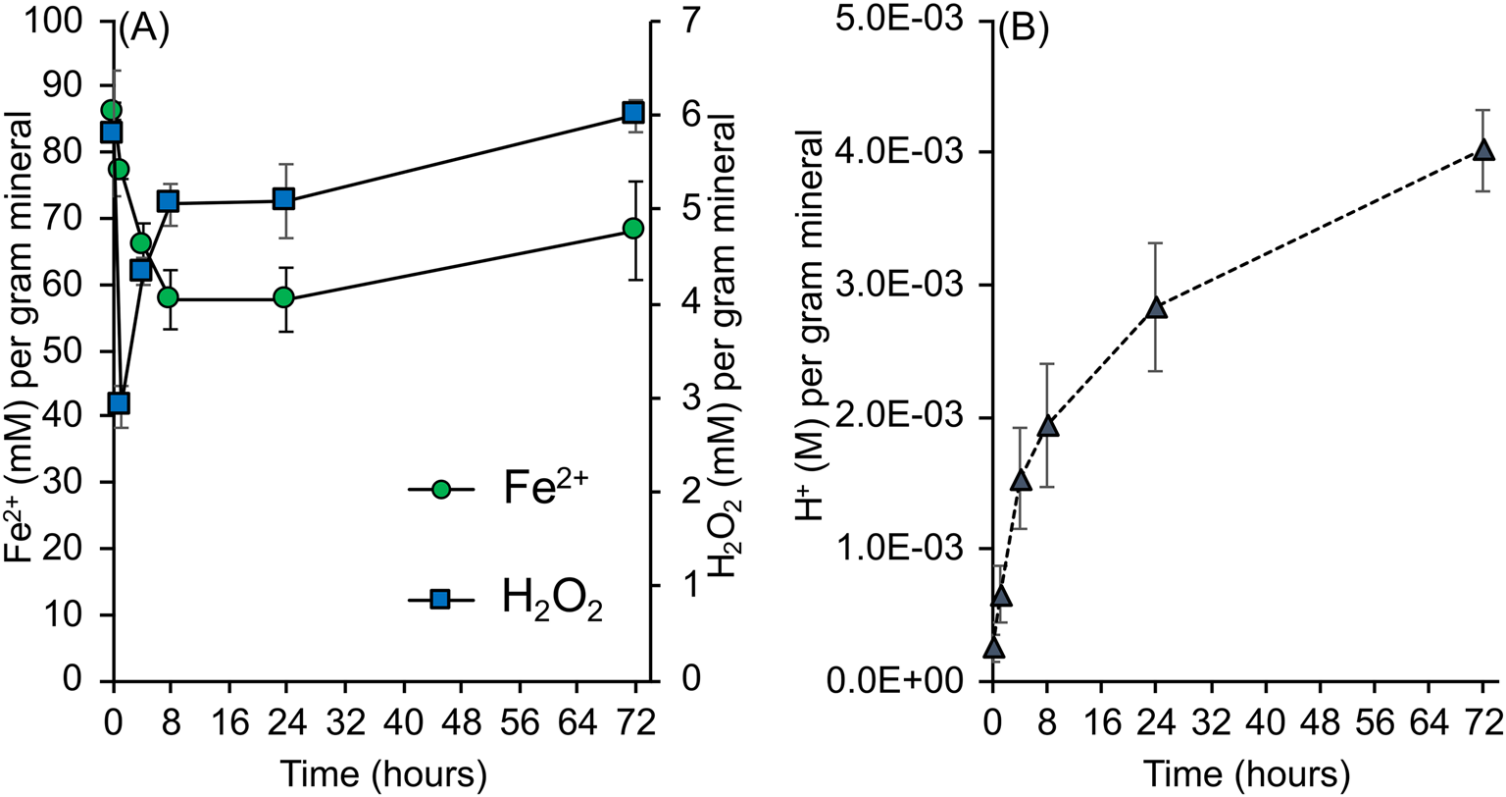
Antibacterial mineral doses of (**A**) Fe^2+^, H_2_O_2_ (mM/g mineral) and (**B**) pH (mM/g mineral) measured over 72 h. All concentrations were normalized to the mass of the minerals used in the suspensions.

The antibacterial mineral formulations released 86.2 mM (± 1.38 mM) Fe^2+^ and 5.79 mM (± 0.67 mM) H_2_O_2_ per gram of minerals when hydrated (Fig. 4A). After 8 h the concentrations of Fe^2+^ fall to 57.7 mM (± 4.6 mM) and stabilize for 24 h. On the third day of reaction Fe^2+^ levels increase to 68.1 mM (± 7.5 mM) (Fig. 4A). The average standard error between all the time points, mineral dose ranges and media solutions was 5.4%, indicating a consistent dose of Fe^2+^ is released per gram of minerals in a range of cation, anion and organic solutions. Reactive oxygen species generation is also an important part of the antibacterial mechanism and the hydrated minerals resulted in the immediate formation of 5.8 mM (± 0.67 mM) H_2_O_2_ per gram of minerals (Fig. 4A). The concentrations of H_2_O_2_ rapidly dropped to 2.9 mM (± 0.22 mM) during the first hour of reaction, followed by an increase in H_2_O_2_ generation to 5.05 mM (± 0.21 mM) after 8 h. Similar to the Fe^2+^ release from 8 to 24 h, the H_2_O_2_ concentrations stabilized and then increased to 6.0 mM (± 0.17 mM) after 3 days (Fig. 4A). The pH measurements also included a 400 mg/mL dose in the averaged measurements. The concentrations of H^+^ released form the hydrated minerals reached mM concentrations (per gram of mineral) after 4 h of reaction (Fig. 4B). The concentrations of H^+^ increased 2.6 times from 4 to 72 h, and maintained mM concentrations of H^+^ in a range of buffering solutions. These results show that a range of mineral doses can overcome solutions with varying pH buffering capacity, cation, anion content and organic composition.

**Figure 5:**
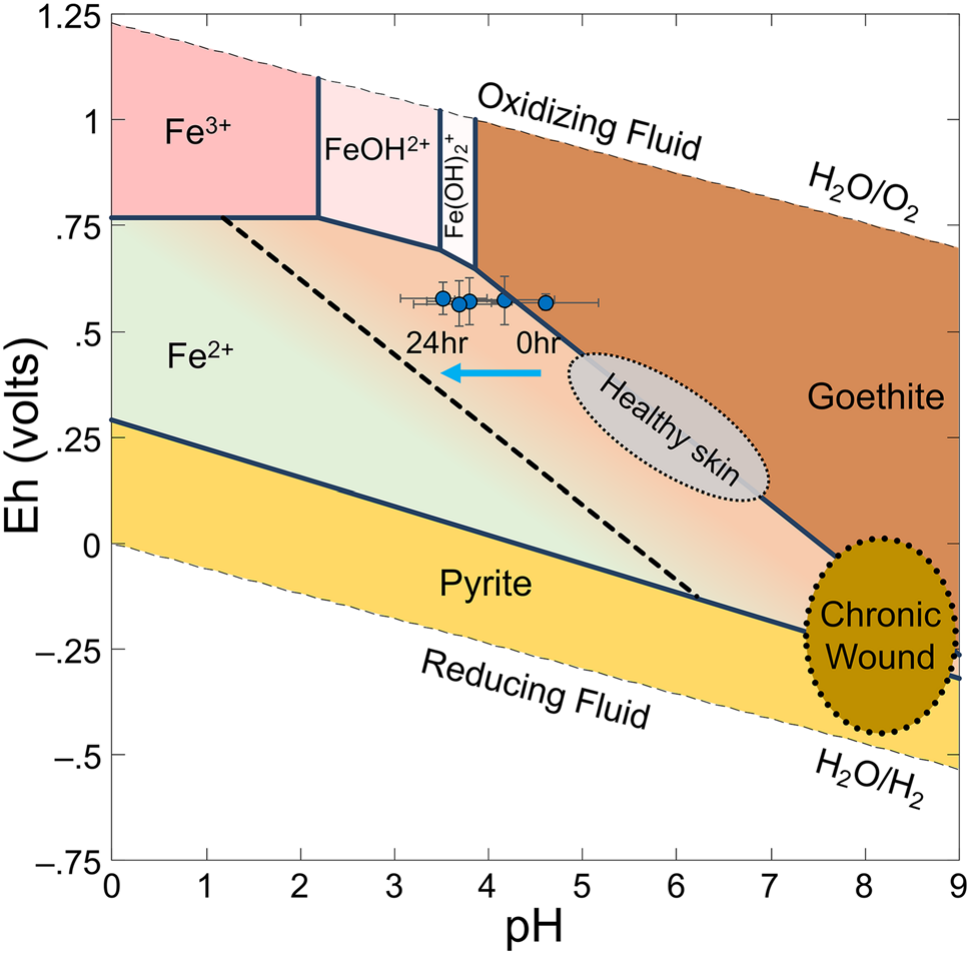
Speciation modeling of the Fe–S–O–H mineral and soluble ion system. Data from antibacterial mineral pH and Eh measurements are overlain and show that the synthetic minerals maintain a low pH oxidizing environment in a range of buffered growth media over 24 h. The pH and Eh data represent mineral concentrations ranging from 50 to 400 mg/mL. The Fe^2+^ stability field is separated by a dashed line that represents the shift in goethite stability that occurs as Fe concentrations increase as a result of pyrite oxidation and smectite interlayer cation exchange.

The average pH and Eh values for mineral doses (50, 75, 100 and 400 mg/mL) were measured over 24 h and plotted against a Fe–S–O–H speciation and mineral stability diagram. The average pH of the antibacterial mineral suspensions decreases from 4.6 to 3.5 over 24 h (Fig. 5). The Eh values range from 553 to 567 mV over 24 h, the changes observed in this time period were not statistically significant. The results reveal the synthetic antibacterial minerals maintain a low pH and oxidizing environment unlike the more reducing alkaline conditions found in a chronic wound. These conditions promote wound healing (Fig. 5). Pyrite (FeS_2_) forms in reducing environments over a wide pH range (Fig. 5). When pyrite is taken out of a reducing environment and placed in oxidizing conditions (in equilibrium with the atmosphere) oxidation reactions occur until a new oxidizing equilibrium is reached^34,37^. The oxidation of pyrite can occur via O_2_ or Fe^3+^ in solution^37^, with iron oxide minerals (goethite) ultimately representing the stable mineral end members (Fig. 5).

### Relevance to clinical wound care

**Figure 6:**
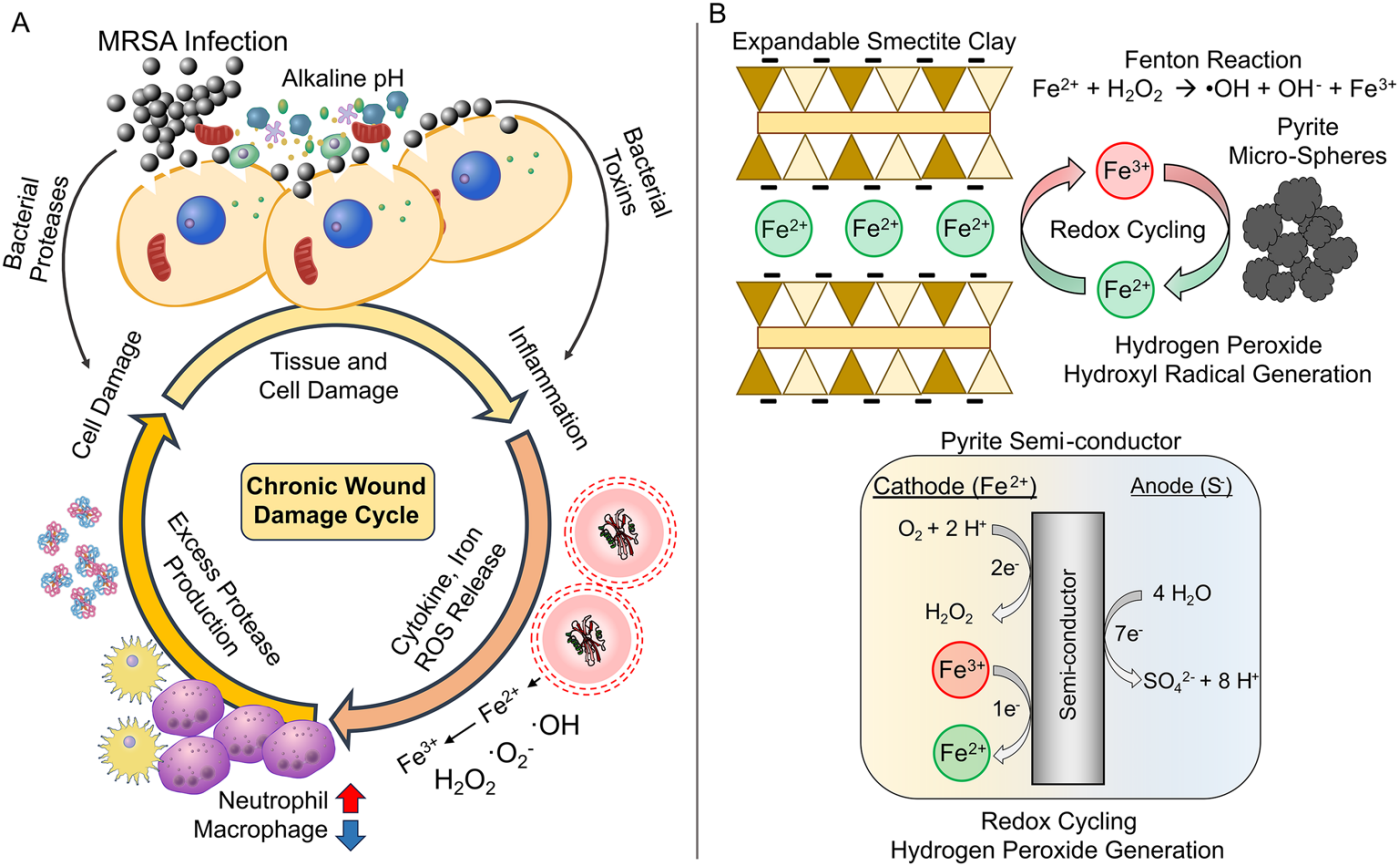
The chronic wound damage cycle and synthetic antibacterial minerals. (**A**) Tissue damage from bacterial infections result in the release of toxins and virulence factors that breakdown host cells, releasing cytokines and iron that cause inflammation. Neutrophil and macrophage production is dysregulated due to the excess production of proteases, which results in further cell damage and a continued damage cycle. (**B**) Synthetic antibacterial minerals establish a redox cycle between Fe^2+^ and Fe^3+^ that is maintained through cation exchange reactions on the negatively charged smectite clay surfaces and direct interactions with the semi-conducting surfaces of the pyrite minerals. The pyrite semi-conductor can be oxidized by O_2_ to produce H_2_O_2,_ or Fe^3+^ to release Fe^2+^ into solution. The acute toxicity from the synthetic antibacterial minerals causes toxic levels of Fe^2+^ and ROS that kill the pathogenic biofilms and break the chronic wound cycle. Chronic wound damage cycle modified from Cullen et al., 2022 and pyrite semiconductor reactions modified from Schoonen et al., 2010.

These findings are important when the clinical application of antibacterial minerals is considered. The fluids in chronic and acute wounds differ in their pH, bacterial load, electrolytes, proteins, inflammatory mediators, proteases, neutrophils, macrophages and platelets^23,24,26,28,29,46–49^. Chronic wounds infected with bacteria tend to exhibit elevated pH levels that prevent the release of oxygen from hemoglobin and lead to wound sepsis^46–50^. Additionally, these bacterially laden wounds can enter a cycle that prevents wound healing. This cycle is marked by host cell and tissue damage by bacterial toxins and proteases, followed by the release of cytokines, iron and ROS^24–27^. The pro-inflammatory cytokines cause a state of chronic inflammation and neutrophil derived proteases that lead to more cell breakdown and inflammation (Fig. 6). Typically, the burst of reactive oxygen species from the host immune system (neutrophils and macrophages) will aid in the eradication of invading pathogens and signal the activity of tissue repairing cells^23–27^. However, the elevated pH of the wound prevents the release of oxygen from hemoglobin, leading to hypoxic tissues that make proper immune system function difficult. This cycle of bacterial tissue damage, inflammation, ROS and protease release leaves the wound in a state of disrepair, making treatment especially complicated when antibiotic resistant bacteria are present (Fig. 6).

Lower healing rates in chronic wounds have also been associated with increased levels of soluble iron in wound fluids relative to acute or healing wounds^28,29,31,51^. The elevated levels of iron lead to the generation of reactive oxygen species in the wound environment that exacerbate the inflammatory response and feeds into the chronic wound cycle^23–25,28,29^. The concentrations of iron in chronic leg ulcer wound fluids show higher concentrations of free iron when compared to normal human plasma, or acute wounds^31^. However, this trend was only observed when iron concentrations were normalized to protein content in the wound fluids. Other studies have found no major differences in total iron concentrations in chronic wound fluids but do observe increased concentrations of ferritin and decreased transferrin in chronic wounds^25,29^. Ferritin is a protein that stores iron in cells, while transferrin is a blood plasma protein that transports iron, bacterioferritins are also present in the wound environment^52,53^. These proteins store iron in the Fe^3+^ oxidation state with the oxidation of Fe^2+^ to Fe^3+^ in a ferrihydrite mineral core^53^. The reduction of this ferritin bound iron can release the protein bound iron from the ferrihydrite mineral core and promote the generation of ROS and a pro-inflammatory environment^25,29,31,52,53^. This cycle of inflammation, increased wound iron levels, ROS, decreased macrophage activity and increased protease activity appear to be driven in part by the increased generation of antioxidants in the chronic wound environment^23,29^, which would provide a pathway to reduce the Fe^3+^ bound in ferritin leading to the release of Fe^2+^ and the propagation of the Fenton reaction resulting in H_2_O_2_ and •OH radical formation.

The total free iron concentrations in the chronic wound environment have been measured at concentrations ranging from 50 to 180 µM^29,31^. When coupled to bacterial virulence factors related to iron scavenging (cell lysis, siderophore release) and increased protease activity, the concentrations of free iron in a chronic wound infected with a bacterial biofilm provide an ample supply of iron for bacterial growth, ROS generation and inflammation^24,29,31,51^. Many studies have attempted to chelate this free iron pool by using iron chelating siderophores that are not preferred by pathogenic bacteria^30,54,55^. However, iron chelation therapy for the treatment of bacterial infections can produce variable outcomes and is not typically a strategy used to routinely treat chronic non-healing wounds with biofilm infections^26,54^.

No harmful effects on uninfected wounds or intact skin were observed, but one limitation of our study is that we recorded observation only for 8 days. Our study reveals that providing a pathogenic biofilm infection with excess soluble Fe^2+^ can actually lead to bacterial cell death and promote wound closure. This concept seems counterintuitive when compared to the chronic wound inflammation, protease, iron release and ROS cycle. However, the concentrations of Fe^2+^ and ROS released by our synthetic antibacterial mineral formulations are 150 times greater than the levels of free iron typically observed in a chronic wound. This would lead to a period of acute Fe and ROS toxicity that lowers the pH of the wound environment while saturating bacterial cells with Fe^2+^ and oxidizing conditions. Previous studies of natural and synthetic antibacterial minerals indicate that bacterial cells are killed via the oxidation of multiple cellular components (DNA, proteins, lipids) as the pathogens rapidly uptake the soluble Fe^2+^ to levels that result in the precipitation of intracellular iron oxide nano-particles^32–36,40,55^. Iron represents a limiting nutrient for bacterial growth and pathogens will secrete virulence factors to breakdown host cells and acquire iron^53^. Ferric iron (Fe^3+^) uptake is tightly regulated by pathogens via the ferric uptake regulator (Fur)^53^, which prevents the buildup of toxic levels of intracellular iron. However, pathogenic bacteria do not typically encounter free soluble Fe^2+^ as the host cells retain both Fe^3+^ and Fe^2+^ in proteins^53,56,57^. The uptake of Fe^2+^ by pathogens is regulated by the Feo system and is controlled by oxygen availability, with expression levels increasing under anerobic environments. Chronic wounds infected with biofilms shift to alkaline pH (7.5–9), which makes the release of oxygen form hemoglobin difficult and causes sepsis^46–49^. This creates a scenario where bacterial biofilm cells in direct contact with skin cells may experience anoxic conditions and up regulate the *feo* operon to facilitate Fe^2+^ uptake^56–58^. Supplying the pathogen with an ample supply of Fe^2+^ when they are in an upregulated Feo state may lead to the rapid uptake of Fe^2+^ and lead to the precipitation of intracellular nano-particles and ROS stress in the cell. This period of rapid acute Fe^2+^ and ROS toxicity will ultimately damage the host cells in the infection, however these negative effects are offset by clearing the bacterial infection and disrupting the chronic wound cycle, allowing wound closure to occur (Fig. 6). Therefore, the concept of ‘iron overload’ to treat bacterial infections should not be overlooked (Fig. 6). Our results show that distinct geochemical reactions can maintain the release of Fe^2+^ and ROS in the wound environment that produces a bactericidal effect. Maintaining this reaction and extended release thru the application of Fe^2+^ and H_2_O_2_ alone is not possible as the Fe^2+^ reacts too rapidly to achieve an antibacterial effect at comparable metal concentrations^41^. These findings reveal synthetic antibacterial mineral systems can be an effective alternative to treat topical biofilm infections when antibiotics fail, opening the door for future clinical applications.

## Data Availability

The datasets used and/or analyzed during the current study available from the corresponding author on reasonable request.
